# Short-term calorie restriction enhances DNA repair by non-homologous end joining in mice

**DOI:** 10.1038/s41514-020-00047-2

**Published:** 2020-08-14

**Authors:** Zhonghe Ke, Denis Firsanov, Brianna Spencer, Andrei Seluanov, Vera Gorbunova

**Affiliations:** Department of Biology, University of Rochester, Rochester, NY 14627 USA

**Keywords:** Molecular biology, Medical research

## Abstract

Calorie restriction (CR) improves health, reduces cancer incidence and extends lifespan in multiple organisms including mice. CR was shown to enhance base excision repair and nucleotide excision repair pathways of DNA repair, however, whether CR improves repair of DNA double-strand breaks has not been examined in in vivo system. Here we utilize non-homologous end joining (NHEJ) reporter mice to show that short-term CR strongly enhances DNA repair by NHEJ, which is associated with elevated levels of DNA-PK and SIRT6.

Calorie restriction (CR) without malnutrition slows the biological aging process and results in lifespan extension in a number of species^1^. In mice, CR by reduction in calorie intake by 30–40% extends both mean and maximum lifespan by 30–40%^2,3^. CR provides many beneficial health effects, including: reduced incidence of tumors, obesity, diabetes, autoimmune diseases, sarcopenia, and cardiovascular diseases^1,4^. CR has also been shown to ameliorate oxidative damage to DNA, protein, and lipids^5–7^. This reduction in oxidative damage has been attributed to a decline in the generation of reactive oxygen species (ROS)^8^, an enhancement of protective mechanisms^9–12^, or an increase in DNA repair capacity^13,14^. Several studies reported that CR prevents age-related decline in base excision and nucleotide excision repair pathways that repair ROS-induced damage to individual bases and UV-induced DNA adducts, respectively^14^ (reviewed in ref. ^15^). The effect of CR on DNA double-strand break (DSB) repair, the pathway that repairs the most severe type of lesion leading to genomic rearrangements, is less studied. DSB repair plays an essential role in the aging process as mutations in multiple genes involved in DSB repair lead to premature aging syndromes and aged tissues accumulate genomic rearrangements consistent with faulty DSB repair (reviewed in ref. ^16^). Long-term CR prevented age-related decline in the main DSB repair pathway, non-homologous end joining (NHEJ), measured using a linearized DNA ligation in vitro assay^17^. CR was also found to prevent age-related decline in the levels of Ku protein, a critical component of NHEJ machinery^18^.

We generated a knock-in reporter mouse that allows to quantitively measure NHEJ efficiency in vivo using I-SceI endonuclease to induce site-specific DNA double-strand breaks within NHEJ reporter cassette integrated in ROSA26 locus^19^ (Fig. 1a). NHEJ reporter cassette^19^ consists of the GFP gene interrupted by the rat Pem1 intron, which does not have homology to the mouse genome, and a “killer” exon flanked by I-SceI sites. GFP is inactivated by the presence of the killer exon in unrearranged construct. I-SceI cuts lead to the release of the killer exon, while successful NHEJ repair re-ligates the intron leading to reactivation of the GFP gene. NHEJ repair events occurring within the intron tolerate deletions (up to 1 kb on each side of the cut) and insertions without interfering with GFP activity.

**Fig. 1:**
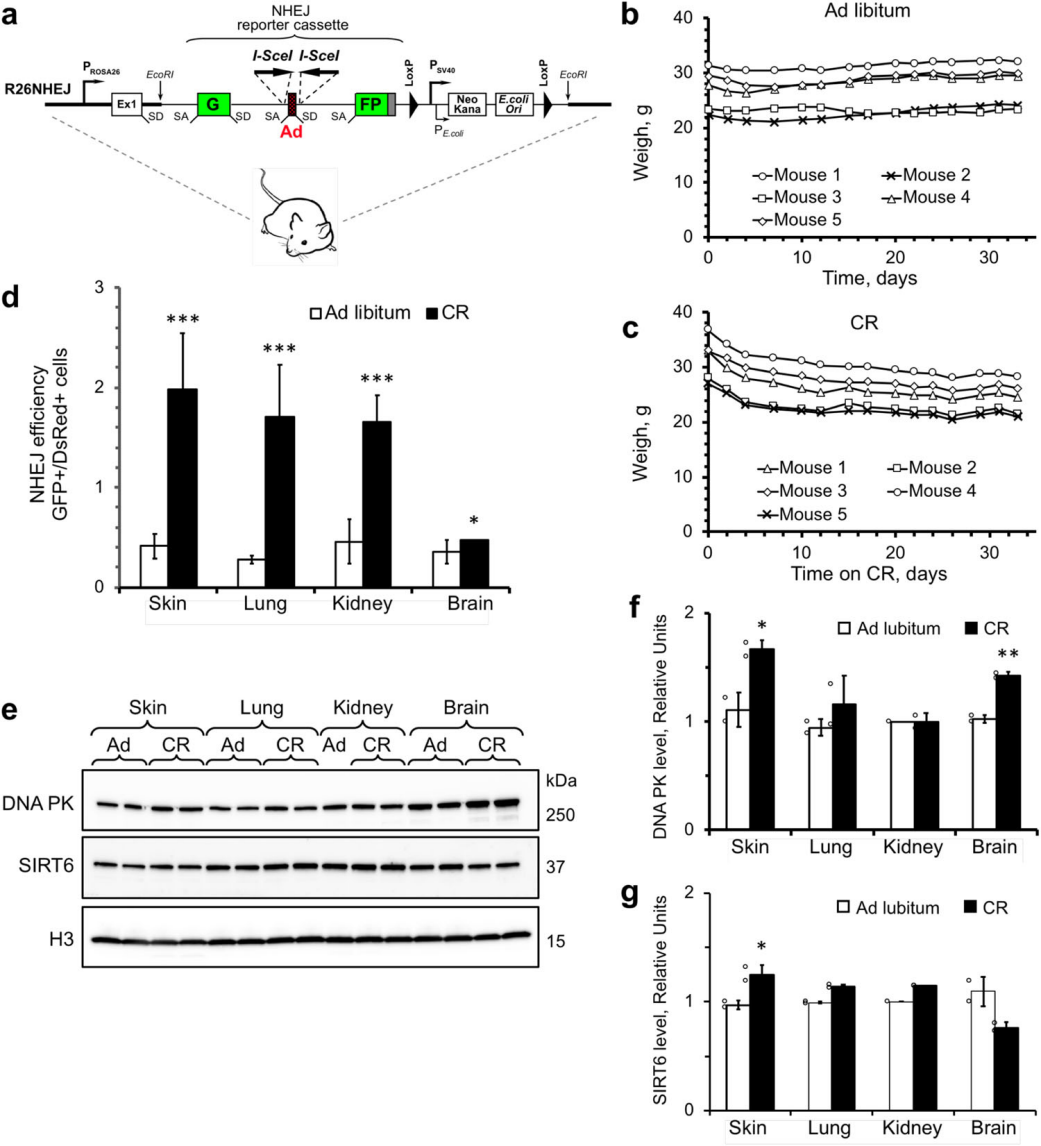
Short-term CR enhances DNA repair by NHEJ. **a** NHEJ reporter cassette integrated in ROSA26 locus. GFP gene is interrupted by an intron containing a “killer” exon that disrupts GFP ORF when spliced in. DSBs are induced by I-SceI endonuclease that cuts on both sides of the “killer” exon generating non-compatible DNA ends. The cuts release the killer exon and NHEJ repair restores the functional GFP gene. **b** Body weight of ad libitum fed mice on precision pellet diet feeding. Mice were fed with at least 50 kcal/day and food pellets were placed on the cage floor. **c** Body weight of the mice on 40% CR. Mice were fed 8.4 kcal/day. **d** NHEJ efficiency is enhanced in CR mice. NHEJ efficiency was analyzed 4 weeks after the start of CR in young adult male mice, five mice per group. Primary cells were isolated from skin, lung, kidney, and brain of the mice, and immediately transfected with the plasmids encoding I-SceI enzyme and DsRed. Representative FACS traces are shown in Supplementary Fig. 1a. **e**–**g** DNA-PK is elevated in the skin, and SIRT6 is elevated in the brain of CR mice. **e** Western blot. **f**, **g** Quantification of the Western blot in **e**. Error bars show s.d., *n* ≥ 3 mice in each group. Significance was calculated using two-tailed *t*-test. *p < 0.05; ***p < 0.001.

The NHEJ reporter mouse model provides a new tool to study the effect of CR on NHEJ efficiency in vivo. Earlier reports did not examine the effect of short-term CR on NHEJ. Notably, it has been reported that 4 weeks of CR is sufficient to ameliorate age-related alterations in DNA methylome^20^, suggesting that short-term CR may be sufficient to enhance DNA repair efficiency. In this report, we used NHEJ reporter mice to examine the effect of short-term 40% CR on NHEJ efficiency. We found that NHEJ efficiency was strongly improved by short-term CR.

To calculate the daily calorie consumption in NHEJ reporter mice, we fed mice ad libitum with dustless precision pellet diet and counted the number of consumed pellets. Mice consumed an average of 14.3 ± 1.3 kcal per day under ad libitum feeding and maintained their body weight (Fig. 1b). In the CR group, mice were fed with 8.4 kcal/day, which was equal to 60% of the average daily calorie consumption. The CR mice lost body weight in the first two weeks of feeding and then maintained their body weight (Fig. 1c). All mice were single housed to ensure equal food consumption. Mice were sacrificed at 4 weeks from the start of the diet. NHEJ was measured in primary cells isolated from fresh skin, lung, kidneys, and brain of the mice. NHEJ assay was conducted as previously described^19^. Briefly, cells were co-transfected with a plasmid encoding I-SceI enzyme to induce DSBs and a DsRed plasmid to normalize for transfection efficiency, and analyzed by flow cytometry. NHEJ efficiency was calculated as a ratio of GFP+ to DsRed+ cells. I-SceI, NHEJ-GFP, and DsRed are expressed from a CMV promoter. Importantly, CR did not alter the intensity of DsRed fluorescence (Supplementary Fig. 1c), suggesting that I-SceI expression was not affected by CR.

We found that short-term CR significantly increased NHEJ efficiency in skin, lung, kidney, and brain tissues, compared to ad libitum-fed mice in skin, lung, kidney, and brain (Fig. 1d). This increase was associated with elevated levels of DNA PK (Fig. 1e, f) and SIRT6 (Fig. 1e, g). These results indicate that short-term CR is sufficient to enhance NHEJ.

Very large deletions may extend into GFP-coding region and result in a loss of signal. Although, theoretically a CR treatment can alter the fidelity of repair, from our previous sequencing studies in AL animals of the same strain, the deletions very rarely extend into the coding region of the GFP gene^19^. Therefore, the observed change in NHEJ efficiency cannot be explained by altered fidelity of NHEJ.

Previous studies showed that CR prevented age-related decline in repair capacity^17,18^, while here we show that short-term CR enhances NHEJ in the young animals. This short-term CR, or perhaps intermittent fasting, may provide added benefit. Environmental stress conferred by CR may trigger hormetic response by activation stress-response pathways, such as SIRT6, and upregulating DNA repair machinery. Improved NHEJ efficiency will result in lower levels of persistent DNA damage, reduced cell death, improved genome stability, and reduced mutation load. This improved genome stability is likely to contribute to lower cancer incidence and longer lifespan conferred by CR. Our finding that even a short-term CR is sufficient to trigger these beneficial effects is important for the practical applications of CR in humans where long-term CR is difficult to achieve, while short-term CR or intermittent fasting is more feasible.

## Methods

### Animals

All mouse experiments were performed in accordance with guidelines established by University of Rochester Committee on Animal Resources. Male mice were used in this study and were all single-housed to perform calorie-controlled feeding experiments. The experiments were performed on 3–5 months old C57BL/6 mice harboring NHEJ reporter cassette in ROSA26 locus generated by Vaidya et al.^19^.

### CR administration

Mice were fed with Dustless Precision Pellet diet (BioServ, Cat# F0074). Calculated amount of precision pellet food was placed on the cage floor every morning, leftover food pellets were counted and removed daily before new food was provided. One mouse became moribund and was discontinued from the CR experiments and excluded for NHEJ analysis.

### NHEJ assay

Primary cell cultures were isolated from skin, lung, kidney, and brain of mice as previously described^19^. One million primary cultured cells were transfected with 5 µg pCMV-I-SceI plasmid to induce DNA DSBs and 0.1 µg pCMV-DsRed plasmid to normalize transfection efficiency, using program T-020 Amaxa Nucleofector II. Mouse cells display high level of auto-fluorescence that must be excluded to obtain reliable data. To avoid the problem of autofluorescence we used very low amounts of DsRed plasmid such that it does not co-inside with the GFP signal allowing us to exclude autofluorescent cells (Supplementary Fig. 1b). Three days post-transfection, cells were harvested and the number of GFP^+^ cells and DsRed^+^ cells was analyzed by flow cytometry. Triplicate transfections were performed for individual primary cultures and NHEJ efficiency was calculated as ratio of GFP^+^ to DsRed^+^ cells.

### Western blotting

Exponentially growing cells were harvested with trypsin, counted and 10^6^ cells were resuspended in 100 µL of PBS containing protease inhibitors. 100 µL of 2×Laemmli buffer (Bio-Rad) was added and samples were boiled at 95 °C for 10 min. Samples were separated with 4–20% gradient SDS–PAGE, transferred to the PVDF membrane, and blocked in 5% milk-TBST for 2 h at room temperature. Membranes were then incubated overnight at +4 °C with rabbit monoclonal antibodies anti-DNA PKcs (Abcam, ab32566, 1:1000), rabbit monoclonal antibodies anti-Sirt6 (CST, #12486, 1:1000) or rabbit polyclonal antibodies anti-Histone H3 (Abcam, ab1791, 1:10,000) in 5% BSA-TBST. After three washes for 10 min with TBST, membranes were incubated for 1 h at room temperature with goat anti-rabbit IgG H&L (HRP) (Abcam, ab6721, 1:5000). After three washes with TBST signal was developed with Clarity Western ECL substrate (Bio-Rad). The images were quantified with Image Lab (Bio-Rad). All blots shown were derived from the same experiment and were processed in parallel.

### Reporting summary

Further information on research design is available in the Nature Research Reporting Summary linked to this article.

## Supplementary information

Supplementary Figure 1

Reporting summary

## Data Availability

All data generated or analyzed during this study are included in this published article (and its supplementary information files) or are available from the authors upon a reasonable request.
