# Supplementary material for: Short-term calorie restriction enhances DNA repair by non-homologous end joining in mice
Source: NPJ Aging Mech Dis. 2020 Aug 14;6:9. doi: 10.1038/s41514-020-00047-2 (PMC7427781; doi:10.1038/s41514-020-00047-2)
Supplement: Supplementary file 1 — Supplementary Figure 1 [file 41514_2020_47_MOESM1_ESM.pdf]

Supplementary Information

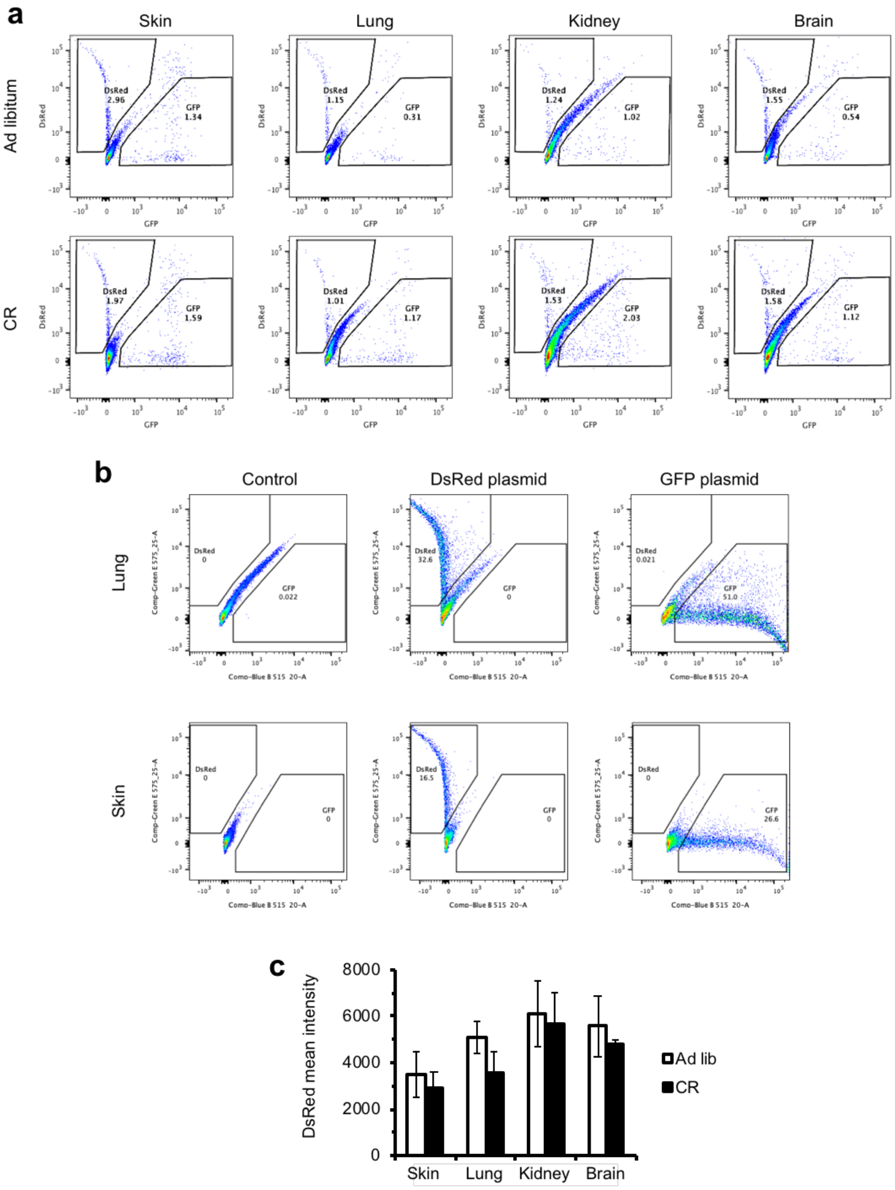

Supplementary Figure 1. Representative FACS traces for NHEJ assay. (a) Primary cells ($1x10^6$) primary cells were transfected with 5 μg I-SceI and 0.1 μg DsRed plasmids in the first passage. Three days after transfection, cells were harvested and analyzed by FACS. NHEJ efficiency was calculated as a ratio of GFP+/DsRed+ cells. (b) The transfection method we used minimizes double transfection, hence the majority of double positive cells are auto-fluorescent

rather than truly double positive as evidenced by untransfected controls. (c) DsRed fluorescence intensity is not altered by CR. Mean fluorescence intensity provided by transfection control DsRed plasmid was calculated for all samples analyzed for NHEJ.

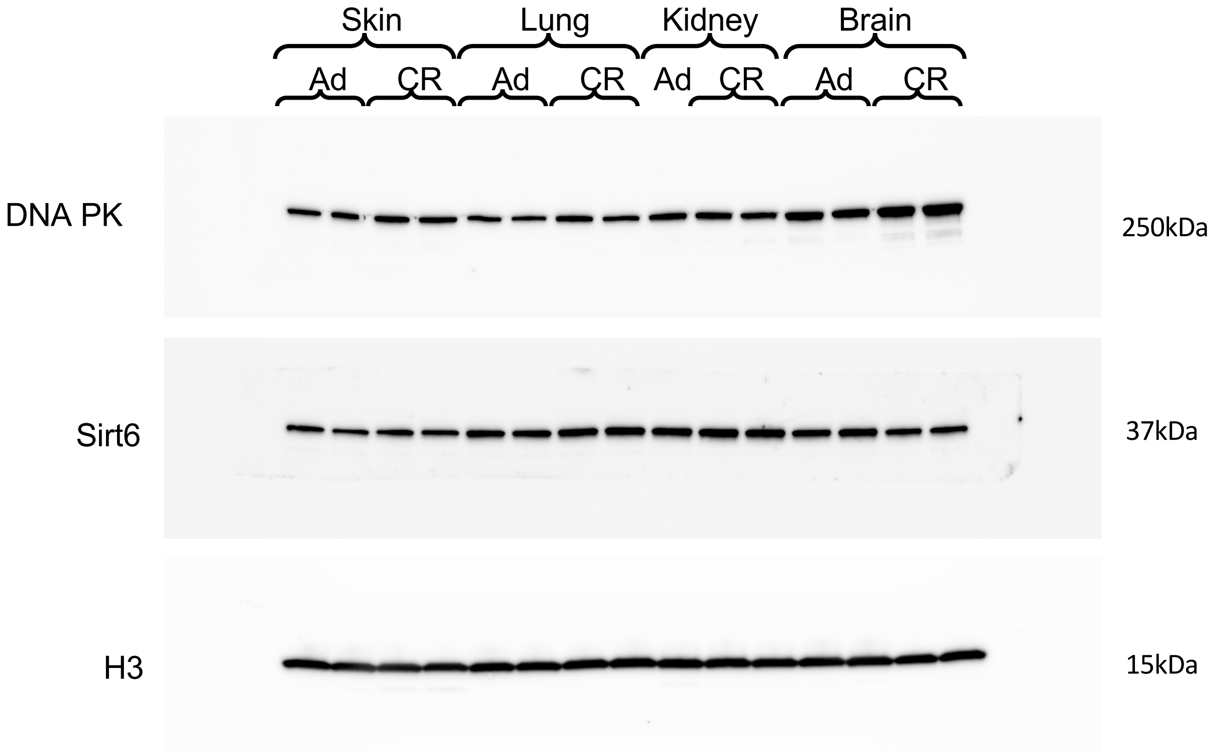

Supplementary Figure 2. Uncropped Western blots.
