# Supplementary material for: Short-term calorie restriction enhances DNA repair by non-homologous end joining in mice
Source: NPJ Aging Mech Dis. 2020 Aug 14;6:9. doi: 10.1038/s41514-020-00047-2 (PMC7427781; doi:10.1038/s41514-020-00047-2)
Supplement: Supplementary file 2 — Reporting summary [file 41514_2020_47_MOESM2_ESM.pdf]

nature research

Corresponding author(s): Andrei Seluanov, Vera Gorbunova

Last updated by author(s): Jul 19, 2020

Reporting Summary

Nature Research wishes to improve the reproducibility of the work that we publish. This form provides structure for consistency and transparency in reporting. For further information on Nature Research policies, see our Editorial Policies and the Editorial Policy Checklist.

Statistics

For all statistical analyses, confirm that the following items are present in the figure legend, table legend, main text, or Methods section.

n/a	Confirmed	
☐	☒	The exact sample size (*n*) for each experimental group/condition, given as a discrete number and unit of measurement
☐	☒	A statement on whether measurements were taken from distinct samples or whether the same sample was measured repeatedly
☐	☒	The statistical test(s) used AND whether they are one- or two-sided<br>*Only common tests should be described solely by name; describe more complex techniques in the Methods section.*
☐	☒	A description of all covariates tested
☒	☐	A description of any assumptions or corrections, such as tests of normality and adjustment for multiple comparisons
☐	☒	A full description of the statistical parameters including central tendency (e.g. means) or other basic estimates (e.g. regression coefficient) AND variation (e.g. standard deviation) or associated estimates of uncertainty (e.g. confidence intervals)
☐	☒	For null hypothesis testing, the test statistic (e.g. *F*, *t*, *r*) with confidence intervals, effect sizes, degrees of freedom and *P* value noted<br>*Give P values as exact values whenever suitable.*
☒	☐	For Bayesian analysis, information on the choice of priors and Markov chain Monte Carlo settings
☒	☐	For hierarchical and complex designs, identification of the appropriate level for tests and full reporting of outcomes
☒	☐	Estimates of effect sizes (e.g. Cohen's *d*, Pearson's *r*), indicating how they were calculated

Our web collection on statistics for biologists contains articles on many of the points above.

Software and code

Policy information about availability of computer code

Data collection	N/A
Data analysis	N/A

For manuscripts utilizing custom algorithms or software that are central to the research but not yet described in published literature, software must be made available to editors and reviewers. We strongly encourage code deposition in a community repository (e.g. GitHub). See the Nature Research guidelines for submitting code & software for further information.

Data

Policy information about availability of data

All manuscripts must include a data availability statement. This statement should provide the following information, where applicable:

- Accession codes, unique identifiers, or web links for publicly available datasets
- A list of figures that have associated raw data
- A description of any restrictions on data availability

All data generated or analyzed during this study are included in this published article (and its supplementary information files) or are available from the authors upon a reasonable request.

Field-specific reporting

Life sciences study design

All studies must disclose on these points even when the disclosure is negative.

Sample size	Sample size of at least 3-5 animals per group was chosen based on our previous experience with NHEJ assays in mice.
Data exclusions	Data were not excluded from analysis. One mouse became moribund and was discontinued from the CR experiments and excluded for NHEJ analysis. For the Western blot, the analysis was limited to the samples where cells remained available after Flow Cytometry.
Replication	Each experimental animal group contained five mice. Each experiment described in the paper were repeated at least 3 times. All replication attempts were successful .
Randomization	Animals were assigned randomly to experimental and control groups.
Blinding	The investigators were not blinded during data collection.

Reporting for specific materials, systems and methods

We require information from authors about some types of materials, experimental systems and methods used in many studies. Here, indicate whether each material, system or method listed is relevant to your study. If you are not sure if a list item applies to your research, read the appropriate section before selecting a response.

Materials & experimental systems

n/a	Involved in the study	
☐	☒	Antibodies
☐	☒	Eukaryotic cell lines
☒	☐	Palaeontology and archaeology
☐	☒	Animals and other organisms
☒	☐	Human research participants
☒	☐	Clinical data
☒	☐	Dual use research of concern

Methods

n/a	Involved in the study	
☒	☐	ChIP-seq
☐	☒	Flow cytometry
☒	☐	MRI-based neuroimaging

Antibodies

Antibodies used	Recombinant Anti-DNA PKcs antibody [Y393] (Abcam, ab32566); SirT6 (D8D12) Rabbit mAb (CST, #12486); Anti-Histone H3 antibody (Abcam, ab1791); Goat Anti-Rabbit IgG H&L (HRP) (Abcam, ab6721)
Validation	Antibodies were validated by the manufacturer:<br>DNA PKcs antibodies: Reacts with: Mouse, Rat, Human, Armenian hamster; Suitable for: WB, IHC-P, ICC/IF<br>Sirt6 antibodies: Species Reactivity: Human, Mouse, Rat, Monkey; Applications: WB, IP, ICC/IF<br>H3 antibodies: Reacts with: Mouse, Rat, Chicken, Dog, Human, Saccharomyces cerevisiae...; Suitable for: CHIPseq, Dot blot, Flow Cyt, IHC-P, Electron Microscopy, ICC/IF, ChIP, IP, WB, ChIP/Chip, ICC

Eukaryotic cell lines

Policy information about cell lines

Cell line source(s)	Primary cell cultures were prepared from skin, lung, kidney and brain of mice.
Authentication	Primary cell cultures were not authenticated.
Mycoplasma contamination	Cell were not tested for mycoplasma contamination but no indication of contamination was observed.
Commonly misidentified lines (See ICLAC register)	No commonly misidentified cell lines were used.

Animals and other organisms

Policy information about studies involving animals; ARRIVE guidelines recommended for reporting animal research

Laboratory animals	The experiments were performed on 3-5 months old male C57BL/6 mice harboring NHEJ reporter cassette in ROSA26 locus generated by Vaidya et al. PLOS Genetics 2014
Wild animals	N/A

Field-collected samples	N/A
Ethics oversight	All mouse experiments were performed in accordance with guidelines established by University of Rochester Committee on Animal Resources.

Note that full information on the approval of the study protocol must also be provided in the manuscript.

Flow Cytometry

Plots

Confirm that:

- [x] The axis labels state the marker and fluorochrome used (e.g. CD4-FITC).
- [x] The axis scales are clearly visible. Include numbers along axes only for bottom left plot of group (a 'group' is an analysis of identical markers).
- [ ] All plots are contour plots with outliers or pseudocolor plots.
- [x] A numerical value for number of cells or percentage (with statistics) is provided.

Methodology

Sample preparation	Cells were dissociated from the plate using trypsin 3 days post transfection, and then pelleted and resuspended in PBS for flow cytometry analysis.
Instrument	BD LSR II
Software	FlowJo 10.6.2
Cell population abundance	Cells were not sorted. The entire population of cells was analyzed.
Gating strategy	Gating strategy is detailed in Supplementary Figure 1. Gating was determined using untransfected cells (autofluorescent cells were excluded), and GFP+ and DsRed+ transfection controls.

- [x] Tick this box to confirm that a figure exemplifying the gating strategy is provided in the Supplementary Information.
